# Palmitic acid, but not high-glucose, induced myocardial apoptosis is alleviated by *N*‑acetylcysteine due to attenuated mitochondrial-derived ROS accumulation-induced endoplasmic reticulum stress

**DOI:** 10.1038/s41419-018-0593-y

**Published:** 2018-05-11

**Authors:** Yang He, Lingyun Zhou, Zhiqiang Fan, Shikun Liu, Weijin Fang

**Affiliations:** 1 Department of Pharmacy, The Third Xiangya Hospital, Central South University, Changsha, Hunan 410013 China; 2 Center of Clinical Pharmacology, The Third Xiangya Hospital, Central South University, Changsha, Hunan 410013 China

## Abstract

Pharmacological inhibition of reactive oxygen species (ROS) is a potential strategy to prevent diabetes-induced cardiac dysfunction. This study was designed to investigate precise effects of antioxidant *N*‑acetylcysteine (NAC) in alleviating diabetic cardiomyopathy (DCM). Echocardiography and histologic studies were performed 12 weeks after streptozocin injection. Protein levels involved in endoplasmic reticulum stress (ERS) and apoptosis were analyzed by western blotting in diabetic hearts or high-glucose (HG, 30 mM)- and palmitic acid (PA, 300 μM)-cultured neonatal rat cardiomyocytes (NRCMs). ROS generation and structural alterations of mitochondria were also assessed. We report that NAC alleviated diabetes-induced cardiac abnormality, including restored ejection fraction (EF %), fraction shortening (FS %), peak E to peak A ratio (E/A) and reduced cardiac hypertrophy and fibrosis. These effects were concomitant with blocked ERS and apoptosis, as evidenced by inactivation of phosphorylated inositol-requiring enzyme-1α (IRE1α)/spliced X-box binding protein 1 (XBP1), phosphorylated protein kinase-like kinase (PERK)/phosphorylated eukaryotic initiation factor 2α (eIF2α) and glucose-regulated protein 78 (GRP78)/activating transcription factor 6 (ATF6α)/C/EBP homologous protein (CHOP) pathways, as well as suppressed Bcl-2-associated X protein (BAX)/B-cell lymphoma-2 (Bcl-2) and cleaved caspase 3 expressions. Mechanistically, PA mediated excessive mitochondrial ROS generation and oxidative stress, which were antagonized by NAC and Mito-TEMPO, a mitochondrial ROS inhibitor. No effects were noted by addition of apocynin, a nicotinamide adenine dinucleotide phosphate (NADPH) oxidase inhibitor, and NADPH oxidase 4 (NOX 4) and NOX 2 expressions were not altered, indicating that PA-induced ROS generation is independent of NADPH oxidases. Most intriguingly, HG failed to promote ROS production despite its ability to promote ERS and apoptosis in NRCMs. Collectively, these findings indicate that NAC primarily abrogates PA-mediated mitochondrial ROS through ERS and therefore alleviates myocardial apoptosis but has little effect on HG-induced cardiac injury. This uncovers a potential role for NAC in formulating novel cardioprotective strategies in DCM patients.

## Introduction

Both type 1 and type 2 diabetes can directly lead to diabetic cardiomyopathy (DCM), which is a major cardiovascular complication that causes mortality and morbidity in diabetes and may be a consequence of mitochondrial dysfunction and myocardial apoptosis^1^, defined as cardiac dysfunction and remodeling in the absence of coronary artery disease and hypertension. Until now, the pathogenesis of DCM has been incompletely understood, and limited treatment options exist.

Endoplasmic reticulum stress (ERS) is an evolutionarily conserved cell stress response and has been associated with DCM. ERS can be activated by oxidative stress and leads to accumulation of unfolded proteins (unfolded protein response (UPR)). The UPR can dissociate ER chaperone glucose-regulated protein 78 (GRP78) from three sensors of ERS to activate them. Inositol-requiring enzyme-1α (IRE1α) can be activated by homodimerization and autophosphorylation after release from GRP78. Activated IRE1α cleaves X-box binding protein 1 (XBP1) messenger RNA (mRNA) to initiate the translation of transcriptionally active spliced XBP1 (sXBP1). Similarly, activating transcription factor 6 (ATF6α) translocates to Golgi from ER and is further cleaved by proteases. A cleaved 50 KDa N-terminal fragment of ATF6α can move into nucleus to induce C/EBP homologous protein (CHOP) expression. Likewise, PERK (protein kinase R-like endoplasmic reticulum kinase) is also activated by homodimerization and autophosphorylation and then phosphorylates α subunit of eIF2α to prevent the formation of translation initiation complexes. Initially, the UPR activates IRE1α, ATF6α and PERK to reduce ER workload by increasing folding and handling efficiency. However, if the UPR fails to reduce ERS and restore homeostasis, ERS causes myocardial apoptosis and cell dysfunction.

ROS contributes to cardiovascular diseases by several mechanisms, including ERS. ROS has been reported to induce ERS in human umbilical vein endothelial cells, which is compromised by addition of ROS scavengers^2^. Another study also supported that ROS mediates ERS and functional impairment of cardiomyocytes in tunicamycin-induced cardiac dysfunction^3^. However, how ROS regulates ERS and apoptosis during the progression of cardiomyopathy under hyperglycemia or hyperlipidemia remains obscure. One of the two main sources of ROS generation is nicotinamide adenine dinucleotide phosphate (NADPH) oxidases, which constitute a family of seven membrane-associated, multiunit enzymes that catalyze the reduction of molecular oxygen using NADPH as an electron donor. It has been demonstrated that only Nox1, Nox2, Nox4 and Nox5 are present in cardiovascular tissues^4^, and upregulated Nox enzymes contribute to oxidative stress and cardiovascular disease^5^. Another source of ROS in cardiovascular cells is from mitochondria as a byproduct of respiration. In particular, complexes I^6^ and III^7^ of the mitochondrial respiratory chain exhibit electron leakage under diabetes that produces superoxide anions. Ni et al.^8^ have confirmed that mitochondrial ROS is increased in cardiomyocytes under diabetic conditions^8^.

*N*-acetylcysteine (NAC) is an antioxidant with ROS scavenging properties that represents a potentially beneficial effect for cardiovascular disease ^9^. Several lines of evidence have indicated that administration of NAC in diabetic mice resulted in improved cardiac fibrosis and inhibited the expression of proinflammatory factors and profibrogenic factors in cardiomyocytes under high-glucose (HG) incubation^10^. NAC also reversed pregestational diabetes-induced oxidative production and decreased the coronary artery volume in fetal hearts of mice^11^, demonstrating the cardioprotective effects of NAC. Consistently, NAC has also been reported to abolish palmitic acid (PA)-induced ERS, total triglyceride (TG) formation and insulin resistance^12^. These studies raise an intriguing hypothesis that the beneficial effect of NAC on diabetic hearts may rely on attenuated ERS and metabolic disorders. However, the precise molecular mechanisms of NAC on DCM in response to high-glucose or high-lipid condition remain unclear.

In the present study, we demonstrate that diabetes induces ROS accumulation and ERS in hearts, which further induces cardiomyocyte apoptosis, whereas all parameters were improved by NAC. The major findings of this study are as follows. (1) Both HG and high fat (e.g., PA) induce ERS and cardiac hypertrophy, whereas HG for 24 h failed to provoke ROS generation, indicating that other mechanisms may be involved in HG-induced ERS in cardiomyocytes. (2) PA-induced ROS generation is mainly from mitochondria but not mediated by NAPDH oxidase. (3) NAC mainly dampens PA-mediated mitochondrial ROS generation and ERS and therefore alleviates high-fat-induced DCM but has little effect on HG-induced ERS. These insights may be useful toward the formulation of novel cardioprotective strategies in patients with DCM.

## Results

### Animal characterization

**Fig. 1:**
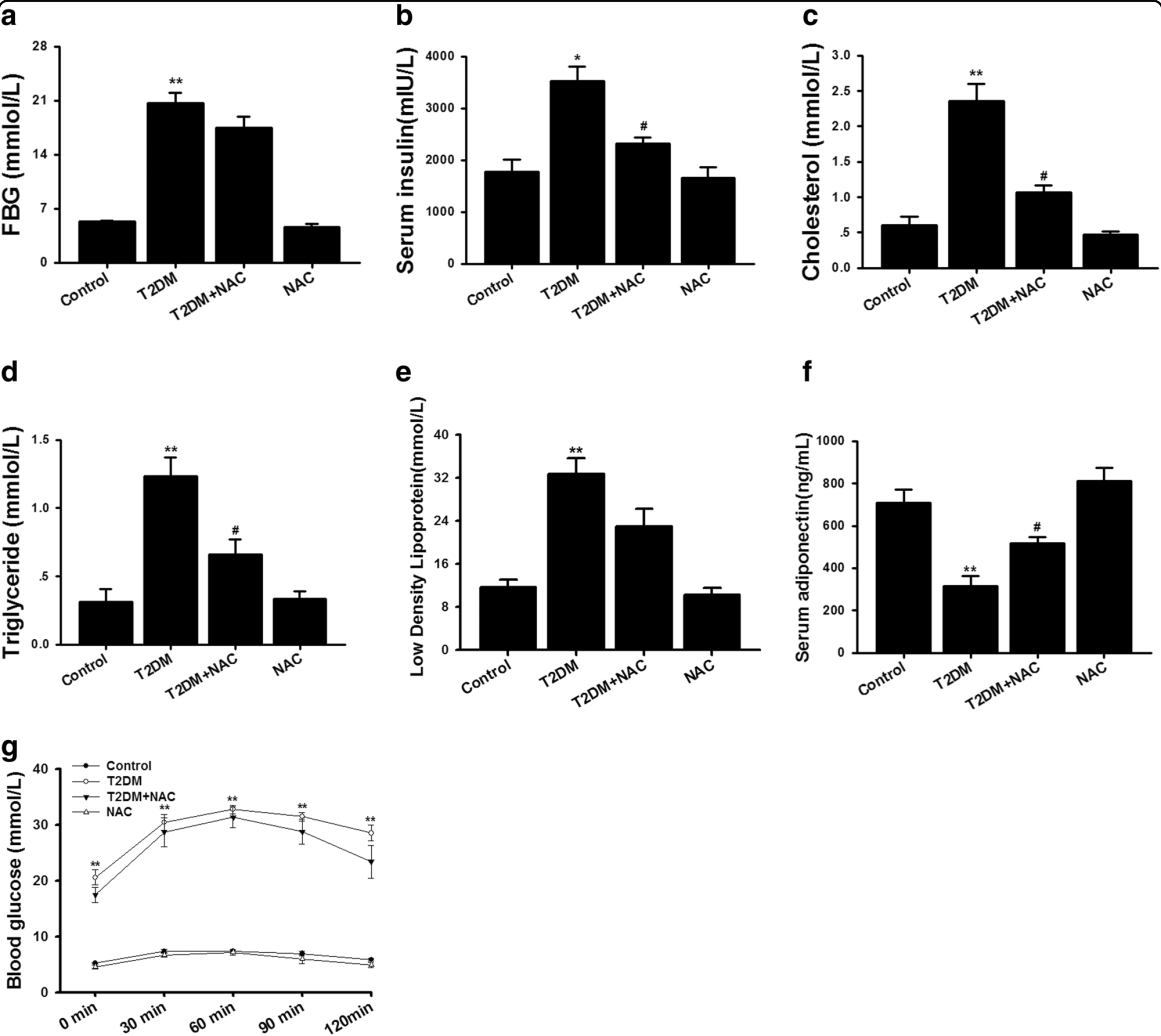
T2DM model displayed hyperglycemia and hyperlipemia. Type 2 diabetic rats model were established by using high-fat diet combined with a single-dose injection of STZ (35 mg/kg) and blood sample were processed for **a** fasting blood glucose levels (FBG), **b** serum insulin levels, **c** cholesterol levels, **d** triglyceride levels, **e** serum adiponectin levels, and **f** low-density lipoprotein levels. **g** Blood glucose concentrations during an oral glucose tolerance test (OGTT) were recorded. Data are presented as the mean±SEM; *n* = 5–8, *P < 0.05, **P < 0.01 vs Control group; ^#^*P* < 0.05 vs T2DM group

We first observed metabolic disorders in an established type 2 diabetic rat model (T2DM). As expected, the model manifested in significantly increased levels of fasting blood glucose (FBG), serum insulin, total cholesterol (TC), TG and low-density lipoprotein (LDL) (Fig. 1a–e), as well as markedly reduced adiponectin (Fig. 1f), compared with control group. In contrast, NAC administration reversed these parameters, with the exception of lowering FBG. An oral glucose tolerance test (OGTT) was also performed at the end of the diet and the data showed that glucose concentrations were dramatically elevated in diabetic rats at all time points (Fig. 1g). Unexpectedly, NAC treatment had no significant affect on blood glucose levels, although it did produce a small but not significant decrease. The blood glucose levels in diabetes and NAC+diabetes groups remained significantly higher than those of control animals. This indicates that NAC has potential to improve lipid disorders but has little effect on glucose homeostasis in T2DM.

### NAC ameliorated cardiac function and structure in diabetic rats

**Fig. 2:**
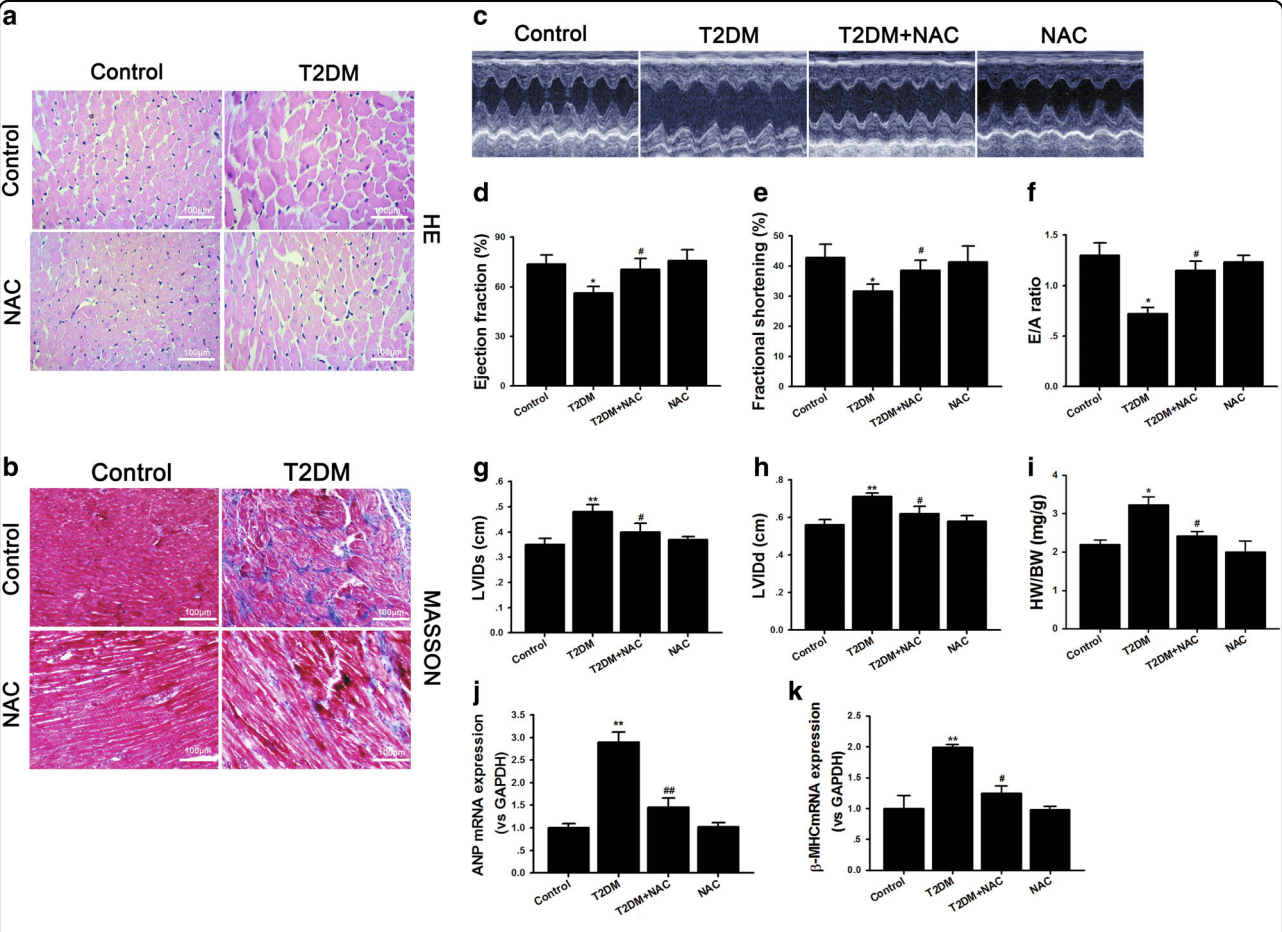
NAC ameliorates the cardiac dysfunction and morphological abnormity induced by type 2 diabetes. **a**, **b** Myocardial hypertrophy and fibrosis were assessed by H&E staining and MASSON trichrome staining in control and T2DM animals with or without NAC administration; scale bar: 100 μm. **c** Transthoracic echocardiography was performed to observe changes in cardiac function and morphology in control and T2DM animals with or without NAC treatment. **d** Evaluation of the ejection fraction (EF %), **e** fraction shortening (FS %), **f** peak E to peak A (E/A) ratio, **g** LV internal dimension at systole (LVIDs), **h** LV internal dimension at diastole (LVIDd) and **i** ratio of heart weight to body weight (HW/BW). **j** Cardiac mRNA expression of atrial natriuretic peptide (ANP) and beta-myosin heavy chain (β-MHC). The data are expressed as the mean±SEM; *n* = 5–8, *P < 0.05, **P < 0.01 vs control group; ^#^*P* < 0.05, ^##^*P* < 0.01 vs T2DM group

We induced cardiac dysfunction and heart remodeling in diabetic rats using high-fat diet combined with a single-dose injection of streptozocin (STZ, 35 mg/kg), a well-established model for DCM in small animals. At 12 weeks after diabetes induction, the rats showed severe cardiac remodeling, including cardiac hypertrophy and interstitial fibrosis detected by hematoxylin and eosin (H&E) and MASSON staining (Fig. 2a, b). Echocardiography showed that diabetes induced significantly more reduction of ejection fraction (EF %), fraction shortening (FS %), and peak E to peak A (E/A) ratio, as well as a greater increase of the left ventricular (LV) internal dimension at systole (LVIDs) and LV internal dimension at diastolic (LVIDd) in diabetic rats (Fig. 2c–h). Consistently, the ratio of heart weight to body weight (HW/BW) was also increased significantly (Fig. 2I). However, NAC administration for 12 weeks protected against cardiac remodeling and cardiac dysfunction caused by diabetes. Similarly, the transcriptional levels of atrial natriuretic peptide (ANP) and β-myosin heavy chain (β-MHC), the fetal genes reactivated during hypertrophy, were both increased more in diabetic hearts, and this effect was also antagonized by NAC treatment (Fig. 2j, k). LV hemodynamic studies were also performed by cannulation of the right carotid artery with a polyethylene Millar pressure catheter to assess cardiac function. The data in Table 1 showed that the diastolic pressure, diastolic duration, systolic duration and cycle duration were all increased, while the mean pressure, pulse pressure and the maximum ascending and declining rate of left ventricular pressure (± dp/dt max) were all reduced significantly in diabetic rats compared to control group, indicating that afterload was increased significantly and left ventricle diastole dysfunction might appear sooner than systolic dysfunction. NAC treatment for 12 weeks ameliorated cardiac function and reduced afterload. These data suggest that NAC is capable of normalizing cardiac hypertrophy and heart dysfunction in a diabetic model.

**Table 1:** Changes of hemodynamic parameters in diabetic rats (*n* = 5–8)

**Parameters**	**Control**	**T2DM**	**T2DM+NAC**	**NAC**
Heart rate (BPM)	393.62 ± 22.07	381.65 ± 20.75	435.93 ± 33.95	424.61 ± 25.33
Systolic pressure (mm Hg)	115.65 ± 10.44	119.24 ± 12.74	109.865 ± 5.24	114.21 ± 0.62
Diastolic pressure (mm Hg)	9.82 ± 0.59	18.08 ± 2.48*	11.05 ± 0.79^##^	7.67 ± 2.64
Mean pressure (mm Hg)	67.57 ± 4.83	44.54 ± 3.99**	56.67 ± 3.00^##^	66.81 ± 4.32
Max–min pressure (mm Hg)	105.97 ± 10.58	65.91 ± 2.62**	97.91 ± 7.11^##^	84.71 ± 8.86
Systolic duration (s)	0.088 ± 0.002	0.11 ± 0.007*	0.088 ± 0.003^#^	0.095 ± 0.008
Diastolic duration (s)	0.082 ± 0.099	0.11 ± 0.006*	0.052 ± 0.008^#^	0.045 ± 0.003
Cycle duration (s)	0.18 ± 0.01	0.21 ± 0.01*	0.14 ± 0.01	0.14 ± 0.003
Max dP/dt (mm Hg/s)	9293.08 ± 113.42	6201.57 ± 528.34**	8886.77 ± 534.30^#^	9408.49 ± 145.84
Min dP/dt (mm Hg/s)	9367.71 ± 193.34	6055.33 ± 354.38**	8385.71 ± 468.94^#^	9436.65 ± 232.20

Data are presented as mean±SEM and were analyzed using one-way ANOVA

*Max dP/dt* maximal rate of increase of left ventricular pressure, *Min dP/dt* maximal rate of decrease of left ventricular pressure

*P < 0.05, **P < 0.01 vs Control group; ^#^*P* < 0.05, ^##^*P* < 0.01 vs T2DM group

### NAC suppressed cardiac ERS and apoptosis in diabetic rats

**Fig. 3:**
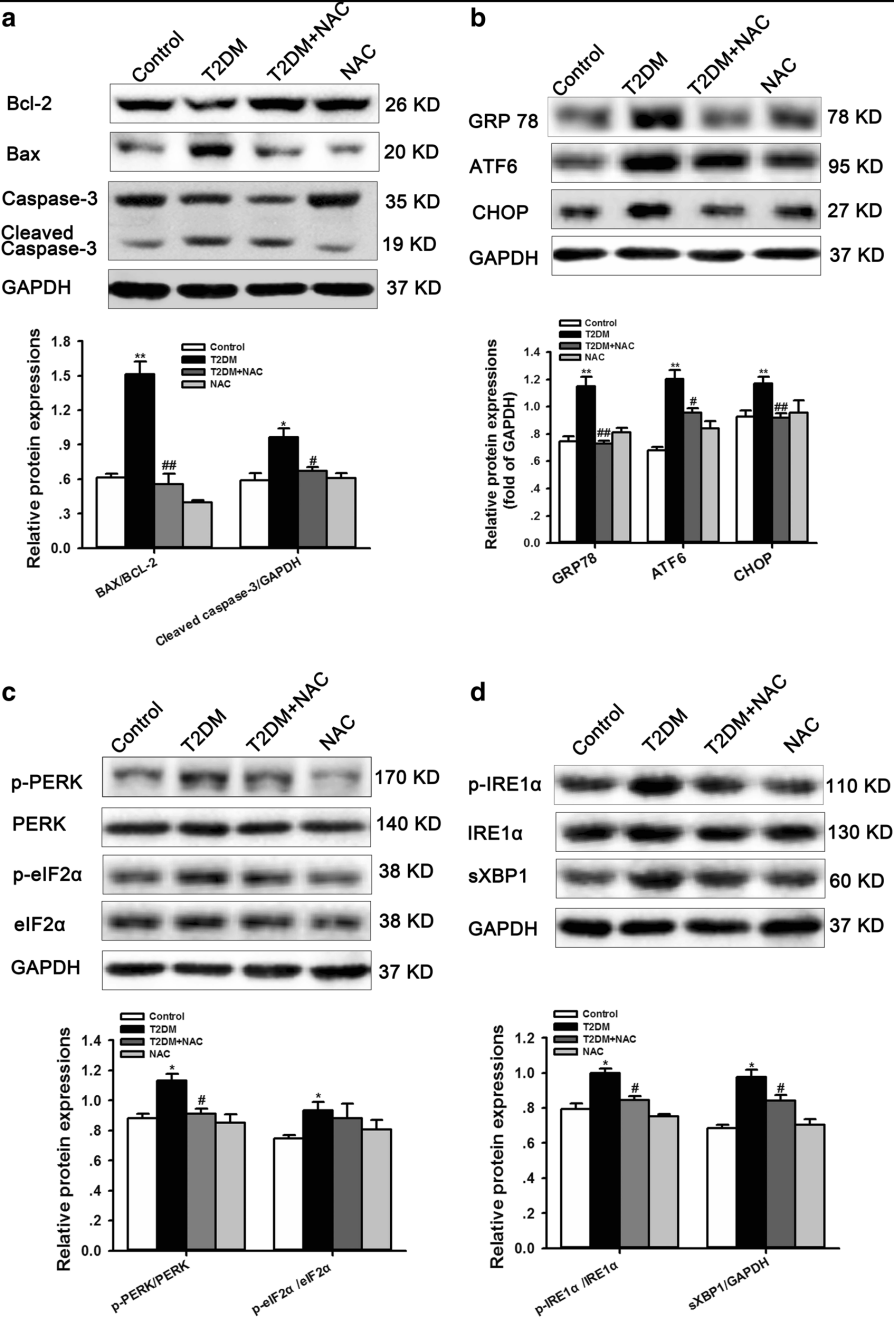
NAC mitigates the apoptosis induced by ERS in diabetic hearts. **a** Representative western blot analysis of Bcl-2, Bax, caspase 3 and cleaved caspase 3. **b**–**d** Representative and quantitative images of the protein expression of ERS markers, including p-IRE1α/ sXBP1, p-PERK/p-eIF2α and the GRP78/ATF6/CHOP pathway. GAPDH was used as a loading control. All values are normalized to the GAPDH band. The data are expressed as the mean±SEM; *n* = 5–8, *P < 0.05, **P < 0.01 vs control group; ^#^*P* < 0.05, ^##^*P* < 0.01 vs T2DM group

Western blot analysis with heart extracts showed that diabetes induced higher levels of Bcl-2-associated X protein (Bax) and cleaved caspase 3 but a lower level of B-cell lymphoma-2 (Bcl-2; Fig. 3a), suggesting that increased apoptosis may be responsible for cardiac dysfunction and remodeling. It is well known that ERS is initiated by UPR. If the UPR fails to reduce ERS and restore homeostasis, ERS causes apoptosis and cell death. To further identify the underlying mechanism by which diabetes induces cardiac dysfunction and remodeling, we analyzed CHOP and ERS-activated signaling pathway effectors including PERK, eIF2α, ATF6, IRE1α, GRP78 and sXBP1. We found that diabetes caused a significant increase in the abundance of p-IREα, sXBP1, p-PERK, p-eIF2α, GRP78, ATF6 and CHOP in diabetic rats, whereas these effects were abolished by treatment with NAC (Fig. 3b–d). No significant differences were found in the abundance of IRE1α, PERK, and eIF2α (Fig. 3c, d). These data suggest that NAC regulates ERS signaling, and the latter may be responsible for increased apoptosis and impaired cardiac dysfunction and remodeling during diabetes.

### NAC alleviated HG- or PA-induced remodeling in cardiomyocytes

**Fig. 4:**
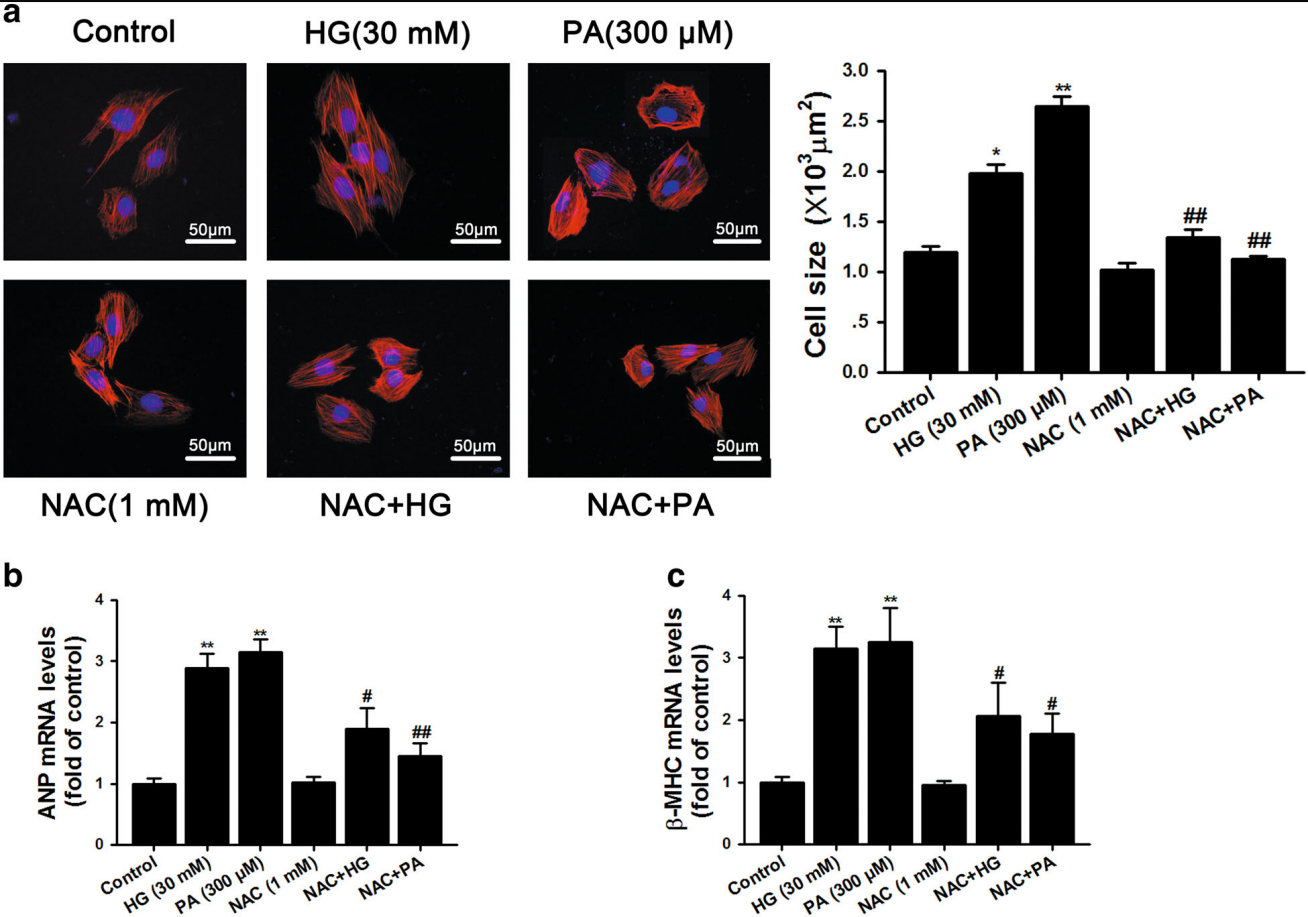
High-glucose- or PA-induced hypertrophic responses were suppressed by treatment with NAC. **a** Cultured NRCMs were incubated with 30 mM glucose or 300 μM PA for 24 h. The cell surfaces were measured by staining with rhodamine-phalloidin; scale bar, 50 μm. **b** The mRNA levels of atrial natriuretic peptide (ANP) were determined by qRT-PCR. **c** The mRNA levels of beta-myosin heavy chain (β-MHC) were determined by qRT-PCR. The results were normalized to GAPDH and presented as the mean±SEM of three independent experiments. *P < 0.05, **P <0.01 vs control group; ^#^*P* < 0.05, ^##^*P* < 0.01 vs group-matched HG or PA-treated NRCMs

We have shown using in vivo experiments that hyperglycemia and hyperlipidemia in diabetes contribute to cardiac dysfunction and remodeling. We next sought to investigate whether HG- or PA-incubated cardiomyocytes show similar remodeling and are therefore representative of T2DM in an in vivo setting. We analyzed cardiomyocyte cell sizes and transcriptional levels of ANP and β-MHC after primary cardiomyocytes were incubated with HG (30 mM) and PA (300 μM) for 24 h. Both HG and PA significantly increased cardiomyocyte cell size (Fig. 4a). Consistent with this, the transcriptional levels of ANP and β-MHC were also remarkably elevated (Fig. 4b, c). All of these changes were ameliorated by incubation with NAC. These results corroborate our in vivo observation that NAC is capable of obliterating HG- or PA-induced cardiomyocyte hypertrophy.

### NAC attenuated PA-induced ERS and apoptosis in cardiomyocytes, but did not have the same effect on HG-treated cells

**Fig. 5:**
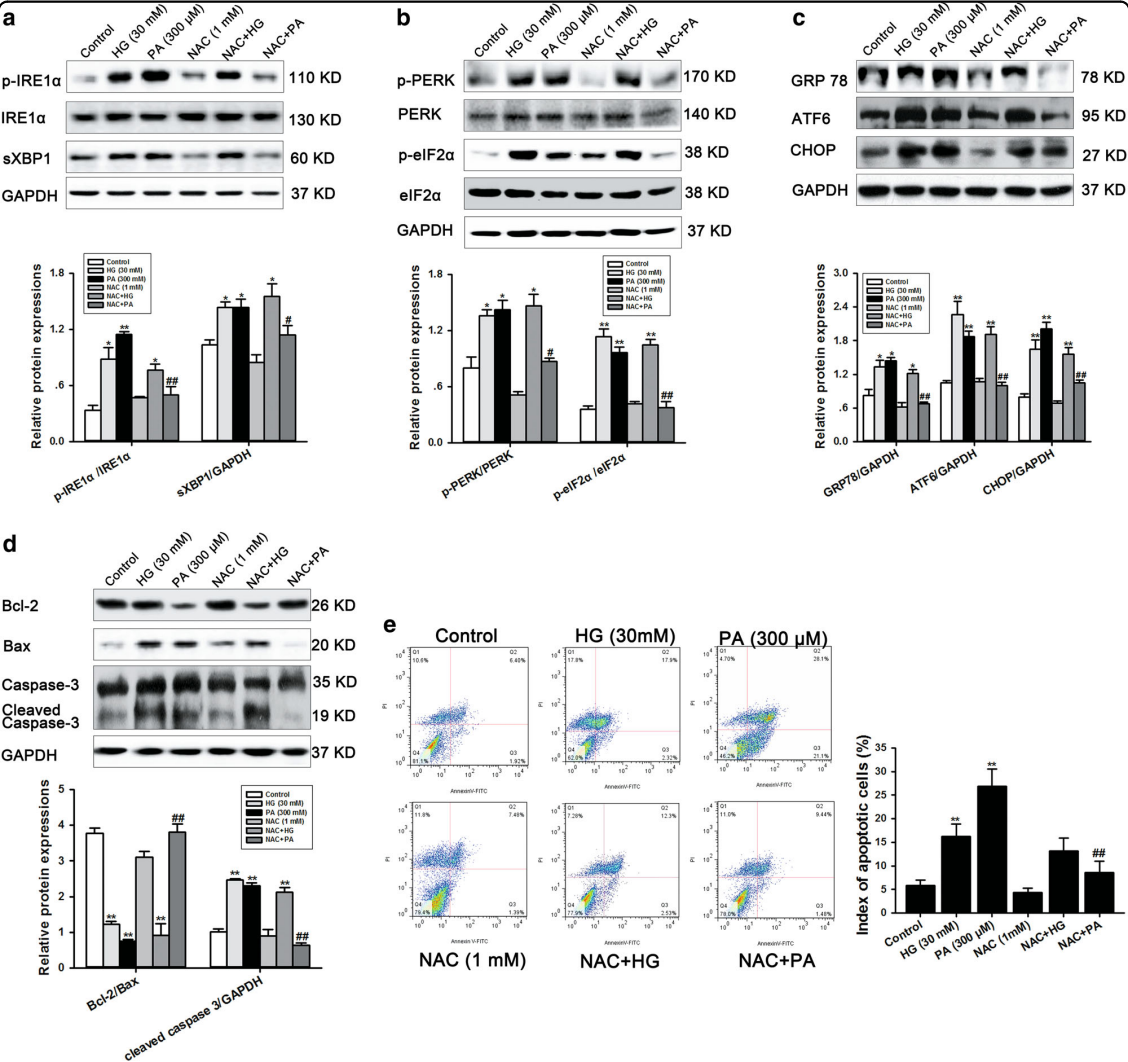
NAC attenuated ERS and apoptosis in PA-treated cardiomyocytes but not in cells treated with high glucose. **a** Representative western blot analysis of Bcl-2, Bax and cleaved caspase 3 in NRCMs after treatment with HG (30 mM) or PA (300 μM) with or without NAC (1 mM). **b**–**d** ERS-associated signaling pathways, such as p-IRE1α/sXBP1 (**b**), p-PERK/p-eIF2α (**c**) and GRP78/ATF6/CHOP (**d**) were also examined by western blotting in NRCMs. GAPDH was used as a loading control. ImageJ software was used to measure the band intensity, and each band intensity was normalized to GAPDH. **e** Annexin V/PI double staining was performed by flow cytometry to detect apoptosis in NRCMs. All data are presented as the mean±SEM of three independent experiments. *P < 0.05, **P < 0.01 vs control group; ^#^*P* < 0.05, ^##^*P* < 0.01 vs PA-treated NRCMs

A large number of studies have shown that HG and PA are sufficient to induce ERS and apoptosis in a variety of cells^13, 14^, which contributes to cell dysfunction. However, the specific effects of NAC on ERS and apoptosis in cardiomyocytes incubated with HG or PA remained unknown. To investigate this, we first confirmed that both HG and PA significantly increased the protein expressions of ERS sensors, such as p-IRE1α, sXBP1, p-PERK, p-eIF2α, GRP78, ATF6 and CHOP (Fig. 5a–c). NAC abolished the pathological effects of PA in cardiomyocytes. No significant differences were found in HG-cultured cardiomyocytes after NAC incubation. Similar to the results observed for ERS, the apoptosis induced by PA was inhibited by NAC, but no effect was found in HG-treated cells (Fig. 5d, e). These results imply that NAC-alleviated diabetes-induced cardiomyopathy rely on inhibiting PA-mediated ERS and apoptosis. In contrast, NAC had no significant effect on HG-mediated ERS and apoptosis, suggesting that different mechanisms are involved in the progression of DCM caused by HG or PA.

### NAC attenuated PA-induced mitochondrial ROS generation and oxidative stress

**Fig. 6:**
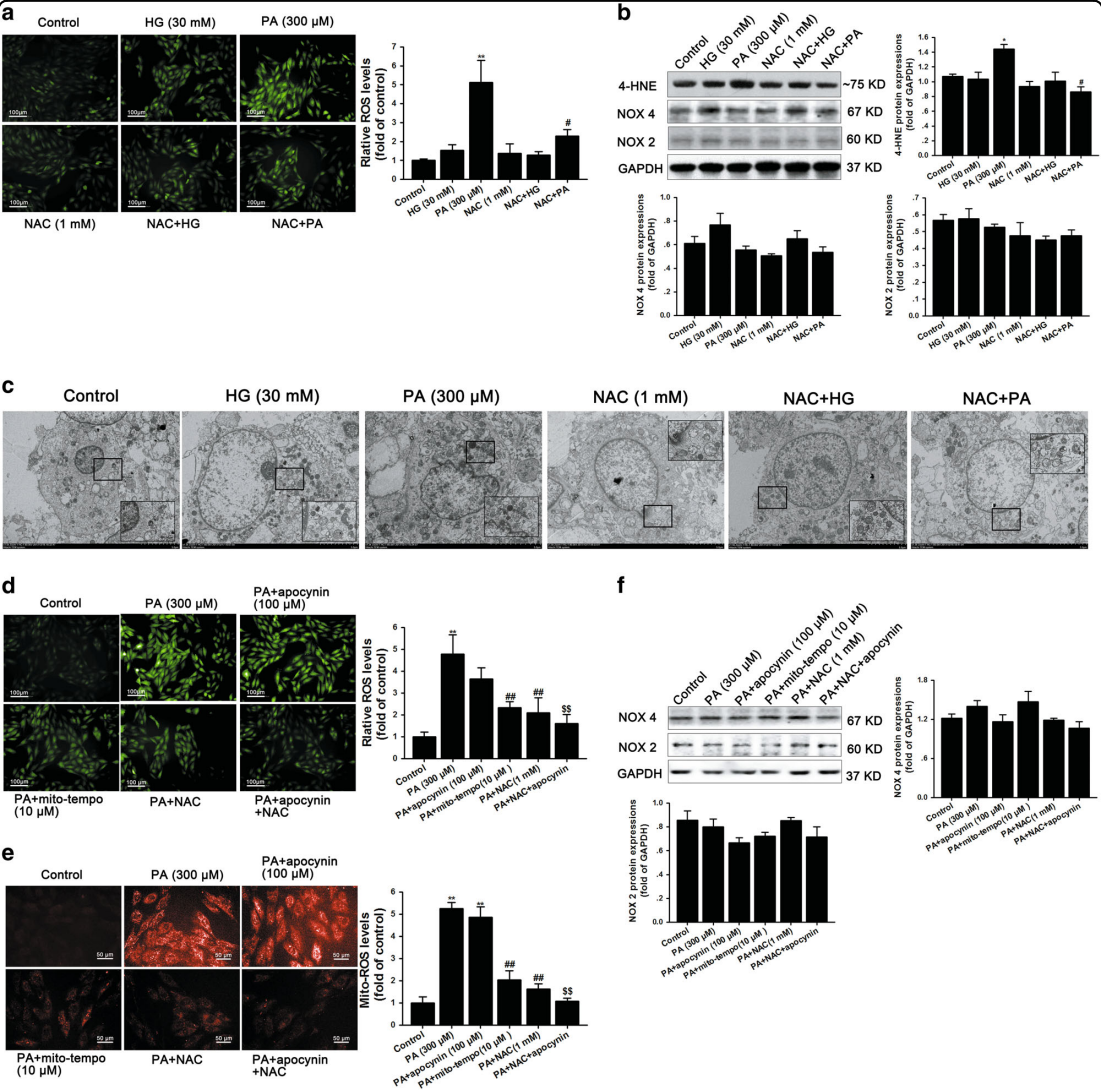
NAC attenuates mitochondrial ROS generation and oxidative stress in cells treated with PA. **a** DCFH-DA staining was performed to determine total ROS levels in NRCMs after being treated with high glucose (30 mM) and PA (300 μM) in the presence or absence of NAC (1 mM) for 24 h; scale bar, 100 μm. **b** 4-HNE, NOX 4 and NOX 2 protein expressions were examined by western blotting in NRCMs. **c** The ultrastructure of NRCMs was observed using a transmission electron microscope (TEM). The black box indicates mitochondria and it was amplified in the rectangle; scale bars: 5 μM. **d** DCFH-DA staining was performed to determine total ROS levels in NRCMs after being treated with PA with or without apocynin (100 μM), Mito-TEMPO (10 μM) and NAC (1 mM) for 24 h; scale bar, 100 μm. **e** Mitochondrial ROS levels were determined by staining with MitoSOX after treatment with PA with or without apocynin (100 μM), Mito-TEMPO (10 μM) and NAC (1 mM) for 24 h; scale bar, 50 μm. **f** NOX 4 and NOX 2 protein expressions were examined by performing western blot after NRCMs were treated with PA with or without apocynin (100 μM), Mito-TEMPO (10 μM) and NAC (1 mM) for 24 h. All western blotting used GAPDH as loading control and ImageJ software was used to measure band intensity and each band intensity was normalized to GAPDH. The fluorescence was imaged and analyzed by using the Operetta® High Content Imaging System. The data were presented as mean±SEM of three independent experiments. ^*^P < 0.05, ^**^P < 0.01 vs control group; ^#^*P* < 0.05, ^##^*P* < 0.01 vs PA-treated NRCMs; ^$$^*P* < 0.01 vs PA+apocynin-treated NRCMs

To clarify whether NAC has similar effects on ROS generation in neonatal rat cardiomyocytes (NRCMs) after treatment with HG or PA for 24 h, we performed fluorescent assays using the fluorescent probe 2’,7’-dichlorofluorescein diacetate (DCFH-DA) to assess total cellular ROS levels. Interestingly, PA (300 μM) remarkably increased ROS levels, whereas HG (30 mM) treatment for 24 h resulted in a slight but not significant increase in ROS generation (Fig. 6a). Consistent with these findings, PA effectively increased the expression of 4-hydroxynonenal (4-HNE), a common byproduct of lipid peroxidation, during oxidative stress. However, HG failed to upregulate 4-HNE expression (Fig. 6b). PA-induced ROS generation and 4-HNE expression were both inhibited by NAC (Fig. 6a, b). These data suggest that 30 mM glucose for 24 h is insufficient to induce oxidative stress in cardiomyocytes.

Excessive ROS generation is mainly due to ectopic activated NADPH oxidases or mitochondrial dysfunction. To delineate the sources of ROS induced by PA, we first observed mitochondrial morphology using transmission electron microscopy, and the data showed that cardiac mitochondria were injured by PA, whereas HG had no significant effect, suggesting that PA-induced ROS generation may result from mitochondrial impairment (Fig. 6c). To further confirm this, we incubated NRCMs with PA in the presence or absence of apocynin, a NAPDH oxidase inhibitor, or Mito-TEMPO, an antioxidant specific for mitochondrial ROS. We first detected the total ROS levels by staining with DCFH-DA, and the result showed that PA dramatically increased total ROS levels, while 100 μM apocynin had a minimal effect on reduction of PA-induced ROS generation. However, ROS generation was reduced by further addition of NAC (Fig. 6d). Similarly, the addition of Mito-TEMPO significantly decreased PA-induced total ROS level (Fig. 6d). To directly elucidate the antioxidant effects of NAC, we further performed mitochondrial ROS staining using MitoSOX. The results indicated that both NAC and Mito-TEMPO negated PA-induced mitochondrial ROS production, whereas apocynin had no effect on this (Fig. 6e). To further confirm this, we performed western blotting to measure the levels of two main isoforms of NADPH oxidase in heart: NOX 4 and NOX 2. The data showed that PA had no significant effect on NOX 4 or NOX 2 expression (Fig. 6f), indicating that PA-induced ROS production is independent of NAPDH oxidases. Together, these observations highlight a significant role for mitochondria-derived ROS specifically in the setting of PA-induced cardiomyopathy, which is specifically inhibited by NAC.

To check whether PA-induced mitochondrial impairment indeed caused cardiomyocytes apoptosis and final cell death, we further used cyclosporin A (CsA), an inhibitor of mitochondrial membrane potential (MMP), to determine its effect on PA-induced cardiomyocytes apoptosis. As expected, both PA and CsA diminished MMP of NRCMs, as shown by reduced JC-1 polymers to monomers fluorescence ratios (Supplementary Figure 1A). However, terminal deoxynucleotidyl transferase dUTP nick-end labeling (TUNEL) assay demonstrated that CsA protected against apoptosis in PA-treated cells (Supplementary Figure 1B). A similar effect was also observed by performing western blotting that CsA markedly reduced elevated ratio of Bax/Bcl-2 and cleaved caspase 3 expressions caused by PA (Supplementary Figure 1C). These data establish a direct relation between mitochondrial impairment and cardiac cell death.

### Mitochondrial ROS contributed to PA-induced ERS and hypertrophy

**Fig. 7:**
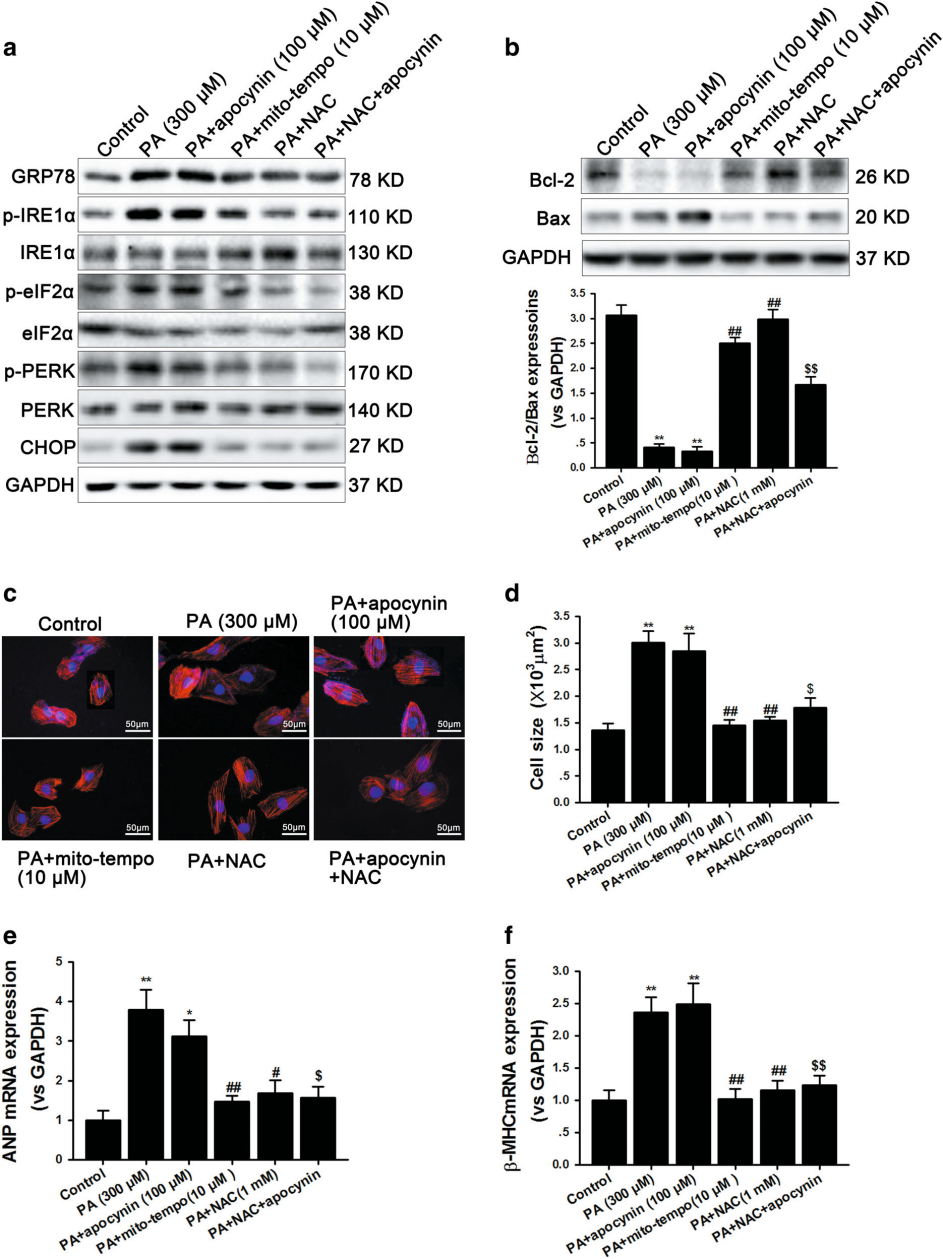
Mitochondrial ROS contributes to PA-induced ERS and hypertrophy. **a** GRP78, p-IRE1α, p-eIF2α, p-PERK and CHOP protein levels were detected by performing western blotting in NRCMs after being incubated with PA (300 μM) with or without apocynin (100 μM), Mito-TEMPO (10 μM) and NAC (1 mM). GAPDH was used as loading control. **b** Western blotting was performed to examine Bcl-2 and Bax expressions to reflect apoptosis. ImageJ software was used to measure band intensity and each band intensity was normalized to GAPDH. **c**, **d** NRCMs were incubated with 300 μM PA for 24 h and then cell surfaces were measured by staining with rhodamine-phalloidin; scale bar, 50 μm. **e** The mRNA levels of atrial natriuretic peptide (ANP) were determined by qRT-PCR. **f** The mRNA levels of beta-myosin heavy chain (β-MHC) were determined by qRT-PCR. All data were presented as mean±SEM of three independent experiments. *P < 0.05, **P < 0.01 vs control group; ^#^*P* < 0.05, ^##^*P* < 0.01 vs PA-treated NRCMs; ^$^*P* < 0.05, ^$$^*P* < 0.01 vs PA+apocynin-treated NRCMs

To further confirm whether PA-induced mitochondrial ROS accumulation is responsible for ERS activation and hypertrophy, we utilized the mitochondrial-targeted antioxidant Mito-TEMPO to cultured NRCMs, followed by analysis of CHOP and other ER stress signaling marker expressions using western blot. Mito-TEMPO obliterated PA-induced more expressions of p-IRE1α, GRP78, p-PERK, p-eIF2α and CHOP (Fig. 7a). Consistently, Mito-TEMPO also protected against apoptosis, as shown by the inhibition of Bax/Bcl-2 ratio. As expected, a similar effect was also observed following addition of NAC, whereas apocynin treatment failed to antagonize PA-induced ERS and apoptosis (Fig. 7b). To further determine whether ERS activation is the main cause of apoptosis, we used 4-phenylbutyrate (4-PBA), a widely used ERS inhibitor, to detect if inhibiting ERS could prevent PA-induced cardiomyocytes death. The data are presented in Supplementary Figure 2 and show that PA-elicited apoptosis was effectively abolished by 4-PBA. Moreover, to specifically examine the role of mitochondrial-derived ROS in PA-induced cardiac hypertrophy, we expose NRCMs to PA for 24 h to induce cellular hypertrophy and measured cell size. The results showed that NRCMs had a less hypertrophic response when treated with Mito-TEMPO and NAC but not with apocynin (Fig. 7c, d). Analysis of levels of ANP and β-MHC also supported this finding (Fig. 7e, f). These results suggest that PA-induced mitochondrial ROS are responsible for ERS activation, and then caused apoptosis, cardiac hypertrophy and final cell death during T2DM.

## Discussion

Effective therapeutic approaches are urgently needed to improve clinical outcomes of patients with DCM. However, despite intense research, the pro-pathogenic factors that can be modulated to prevent DCM remain poorly understood. In particular, the involvement of lipotoxicity, ROS and ERS in this process remains obscure. Our studies using a rat model of type 2 diabetes in vivo and isolated NRCMs in vitro provide the following major findings. (1) PA drove mitochondrial ROS generation to cause ERS, apoptosis and cardiac injury independent of NAPDH oxidase, while HG for 24 h failed to induce mitochondrial ROS production. (2) NAC substantially abrogated PA-mediated mitochondrial ROS generation, ERS and therefore alleviated high-fat-induced DCM but had little effect on HG-induced ERS and apoptosis.

In the current study, we first duplicated essential characteristics of DCM using in vivo experiments, as evidenced by decreased LVEF, LVFS and E/A ratio as well as increased LVIDs and LVIDd by echocardiography, together with decreased±dP/dt max and increased diastolic pressure and cycle duration as assessed by carotid intubation. Additionally, histological observations were also supported by H&E and MASSON staining, as shown by a larger cross-sectional area and intensive interstitial fibrosis distribution in diabetic hearts, which was further confirmed by sharply elevated levels of hypertrophic markers ANP and β-MHC. All of pathological changes in hearts were paralleled with increased levels of blood glucose, triglyceride, cholesterol and LDL, decreased serum adiponectin and impaired glucose tolerance. These data provide in vivo evidence that diabetes-induced cardiomyopathy was established successfully. Although all of pathological parameters in the heart were rescued by treatment with NAC, it is important to mention that NAC did not lower the blood glucose level, and this result is similar to the work of Shen et al.^15^ that NAC intervention for 6 months significantly improved intraperitoneal glucose tolerance test (IPGTT) and insulin tolerance test (IPITT) compared with the high-fat diet (HFD) group. However, NAC intervention for 4 months although showed improved IPGTT and IPITT, it still had impaired IPGTT and IPITT at the end of the study, indicating that the starting time and duration of NAC administration can influence drug effect on insulin sensitivity and blood glucose level. In our present study, NAC intervention 3 months (12 weeks), produce a small but not significant improvement on OGTT in type 2 diabetic rats (more severe than HFD-induced obesity mice), is reasonable. In contrast, dyslipidemia was improved by NAC, prompting us to speculate that NAC-mediated cardiac protection in diabetes is mainly dependent on counteracting the harmful effects induced by lipid overload.

Cardiac lipotoxicity is characterized by increased lipid uptake, incomplete β-oxidation and intermediate accumulation, which is closely related with increased ROS from mitochondria, and contributes to cardiac dysfunction in diabetes. Mitochondrial morphological changes and elevated ROS generation are observed in palmitate-treated NRCMs^16^. Elevated ROS activates the DNA damage response and reduces cardiomyocyte proliferation, resulting in lethal cardiomyopathy. Pharmacological inhibition of ROS restored cardiomyocyte proliferation in cultured fetal cardiomyocytes^17^. These findings enabled us to visualize the important contribution of ROS in DCM. However, diabetes is a complicated and chronic progression characterized by hyperglycemia and hyperlipemia. Thus, elucidating the influence of each risk factor, including high glucose, high lipid and ROS production, helps to purposefully explore precise treatments to prevent the development of diabetes and subsequent cardiomyopathy. In our in vitro experiments, we found that both HG (30 mM) and PA (300 μM) were capable of provoking ERS and apoptosis in NRCMs. Mechanistically, PA administration, at least in this model, mainly increased mitochondrial ROS generation independent of NADPH oxidases, implying that PA-induced ERS and apoptosis may be due to increased ROS and consequent oxidative stress. However, no significant difference in ROS production was noted following HG treatment for 24 h; this result clearly suggests that incubating cardiomyocytes with 30 mM glucose for 24 h is insufficient to induce oxidative stress. Similar data were observed by Wang et al.,^18^ who indicated that treatment with 33.3 mM glucose for 48 h strongly induced ROS generation in cardiomyocytes, while 33.3 mM glucose for 24 h failed to prompt ROS production and apoptosis. The data from our and previous study suggest that the initial mechanisms of HG-induced cardiac ERS and apoptosis are distinct from lipid overload and may not be associated with oxidative stress, as another study reported that NAC could attenuate HG-induced nuclear factor-κB activation and inflammation in H9c2 cells^19^. This finding is somewhat in agreement with the previous notion that only the glucolipotoxic condition of 30 mM glucose in combination with 250 μM palmitate resulted in significant ROS generation, which caused activation of caspase 3^20^. Xia et al.^21^ also reported that NAC prevented hyperglycemia-induced hypertrophy in NRCMs due to blunted PKCβ2 overexpression. Therefore, NAC, which acts as an antioxidant, has no effect on HG-mediated ERS and apoptosis, which may attribute to the inability of HG to induce ROS generation. The other mechanisms involved require further investigation.

Furthermore, we demonstrated that NAC specifically inhibited mitochondrial-derived ROS generation induced by PA. Dai et al.^22^ reported that NAC did not show any beneficial effect on angiotensin-induced ROS generation and cardiomyopathy; this suggests that angiotensin-induced ROS may result from NOX 4 and/or indirectly from amplified NOX 2 isoform. Although Block et al. have reported that NOX 4 may be present in mitochondria in cultured mesangial cells, whether NOX 4 is present in myocardial mitochondria remains controversial. NADPH oxidase must interact with cytochrome b558 at the cytosolic membrane to generate an activated form that catalyzes the transfer of electrons to molecular oxygen, producing superoxide anion^23^. Thus, NADPH oxidase mainly functions in cytoplasm. Consistent with this, our present study supplies convincing evidence that PA-induced mitochondrial ROS is independent of NADPH oxidase, as apocynin had no effect on reduction of ROS levels caused by PA, suggesting a mitochondrial origination. The transmission electron microscopy data also supported this conclusion. Likewise, Byrne et al.^24^ have confirmed that NAC treatment can ameliorate cardiomyopathy induced by acute aortic banding in mice. However, the therapeutic effects of NAC on cardiomyopathy may be also limited by different animal models. Nonetheless, in the present study, NAC attenuated PA-induced cardiomyocyte apoptosis and cellular hypertrophy by inhibiting mitochondrial-derived ROS generation, and this protective effect may be mediated by an ERS-related signaling pathway.

In conclusion, this study demonstrated that cardiac lipid overload specifically stimulates mitochondrial ROS generation to induce apoptosis through ERS, which promotes DCM. Additionally, the cardioprotective effect of NAC relies on attenuated dyslipidemia-induced ERS and myocardial apoptosis via targeted inhibition of mitochondrial oxidative stress. These findings uncover novel prospects for using NAC to develop novel cardioprotective strategies in patients with DCM.

## Materials and methods

### Reagents and antibodies

*N*-acetylcysteine, the mitochondrial ROS inhibitor Mito-TEMPO, apocynin, PA, STZ, CsA, 4-PBA and DCFH-DA were obtained from Sigma-Aldrich (Sigma, St. Louis, MO, USA). MitoSOX Red superoxide indicator was obtained from Molecular Probes (Thermo Fisher Scientific, Waltham, MA, USA). Serum TC, TG and LDL cholesterol assay kits were purchased from Dongou Co., Ltd (Wenzhou, China). The BCA protein assay kit, RIPA lysis buffer and BeyoECL Plus kit were purchased from Beyotime Biotechnology (Shanghai, China). The primers for ANP, β-MHC and glyceraldehyde 3-phosphate dehydrogenase (GAPDH) were synthesized by Invitrogen (Grand Island, NY, USA). Antibodies to cleaved caspase 3 (#9665S), BAX (#5023), Bcl-2 (#15071), PERK (#5683), phosphorylated PERK (#3179), eIF2α (#5324), phosphorylated eIF2α (#3398), ATF6 (#65880), IRE1α (#3294), GRP78 (#3183), sXBP1 (#12782), CHOP (#2895) and GAPDH (#5174) were purchased from Cell Signaling Technology (Massachusetts, USA). Antibodies to eIF2α (phospho S51, ab32157), IRE1α (phospho S724, ab81936), 4-HNE (ab48506), NOX 4 (ab133303) and NOX 2 (ab129068) were purchased from Abcam (Cambridge, UK).

### Animals

All animal studies were performed in accordance with the Care and Use of Laboratory Animals published by the US National Institutes of Health (NIH publication, 8th edition, 2011) and was approved by the Experimental Animal Care and Use Committee of Central South University. Specific pathogen-free male Sprague Dawley (SD) rats weighing 130–150 g were obtained from Hunan SJA Laboratory Animal Co., Ltd (Changsha, China). Upon arrival, the animals were housed in a temperature-controlled room with a 12-h light/dark cycle and four rats per cage were acclimatized for 1 week and fed normal chow ad libitum.

### Experimental protocol

T2DM was induced in SD rats (approximately 8 weeks old) by a single intraperitoneal injection of STZ (35 mg/kg, in 0.1 mol/L citrate buffer, pH 4.5) combined with HFD (sucrose 20%, lard 15%, cholesterol 1.2%, sodium cholate 0.2%, casein 10%, calcium hydrophosphate 0.6%, chow 52.2%). The control groups received normal chow and the same volume of citrate buffer. Whole blood was obtained from the tail vein, and random glucose levels were measured using a glucometer (OneTouch Ultra, USA) 72 hours after STZ injection. Rats were considered diabetic and were used for the study only if they had hyperglycemia (≥16 mmol/L). Diabetic rats continued to consume a high-fat diet for an additional 12 weeks after diabetes confirmation. Rats were divided into four groups: (1) Control group (chow diet, *n* = 5–8); (2) T2DM group (HFD+STZ, *n* = 5–8); (3) T2DM+NAC group (diabetic rats were given NAC (1 mg/mL) through the drinking water^25^, the concentration of NAC was adjusted to give a daily intake of 0.8–0.9 g/kg, *n* = 5–8); and (4) NAC group (age-matched control rats were fed normal chow and received the same amount of NAC, *n* = 5–8). At the end of the experiment, the rats were subjected to the following experiments.

### Echocardiography

Animals were lightly anesthetized with inhaled isoflurane (1%) and imaged with a Vevo 2100 (VisualSonics Inc., Canada) equipped with a 30-MHz transducer. Transthoracic M-mode echocardiograms at the level of the papillary muscles were used to assess changes in the LV internal diameters (LVIDs and LVIDd) and wall thicknesses. The ejection fraction (LVEF %) and shortening fraction (LVFS %) were calculated from M-mode tracing to reflect systolic function. To assess diastolic function, we obtained the pulsed wave Doppler measurements of maximal early (E) and late (A) transmitral velocities (E/A ratio) in the apical view with the cursor at the mitral valve inflow.

### Isolation and culture of neonatal rat primary cardiomyocytes

NRCMs were obtained from 1-day-old neonatal SD rats provided by Hunan SJA Laboratory Animal Co., Ltd (Changsha, China). All experimental procedures and protocols adhered to the NIH Guide for the Care and Use of Laboratory Animals and approved by the Institutional Animal Care and Welfare Committee of Central South University. The procedures were performed as previously described^26, 27^. Neonatal rats were anesthetized by intraperitoneal injection of chloral hydrate (10%, 30 mg/kg) and killed by cervical dislocation; a midline incision was made through the second sternum. The hearts were gently removed and placed in a dish with phosphate-buffered saline (PBS) to wash off the blood. Next, the hearts were cut into 1 mm^3^ pieces and digested with 0.1% collagenase II (Worthington, USA). The enzyme digestion process was performed for 3–5 min each time and repeated approximately 5–7 times at 37 °C until all of the tissue blocks were digested. The digest solutions were collected, and trypsinization was terminated with 10% fetal bovine serum (FBS, Gibco). Then, the cell suspensions were centrifuged at 1000 × *g* for 3–5 min, and pellets were resuspended with Dulbecco’s modified Eagle’s medium containing 10% FBS and incubated for 45 min with differential adhesion to reduce non-myocyte contamination. Afterwards, 0.1 mmol/L 5-bromo-2-deoxyuridine^28^ was added to the remaining cell suspension, followed by culture in 6-well plates. Cardiomyocytes were treated with 5.5 mM glucose, 30 mM glucose (HG) or 300 μM PA. The cells were then harvested for PCR, western blot or cell apoptosis assays.

### Oral glucose tolerance test

OGTT was performed as described previously to evaluate insulin sensitivity^29^. In brief, after overnight fasting, blood samples were collected from the rats’ tail veins to obtain baseline blood glucose levels. Subsequently, the rats in all groups were orally gavaged with 2 g/kg body weight of glucose solution (40%, wt/vol). Blood glucose levels were measured at 0, 30, 60, 90 and 120 min using a glucometer (OneTouch Ultra, USA).

### Heart weight to body weight ratio

At the end of experiment, total body weight (BW) was first obtained, and then the rats were killed and the hearts were harvested, dried and weighed. The cardiac hypertrophy index was assessed by measuring the ratio of the heart dry weight to the total body weight (HW/BW). Then, the hearts were frozen in liquid nitrogen and stored at −80 °C until use.

### H&E and MASSON staining

The pathological changes in the heart tissue of the diabetic rats were observed using H&E and MASSON staining. The heart tissues were fixed in 4% paraformaldehyde^30^ and then dehydrated, embedded in paraffin and cut into 3 μm sections. The sections were deparaffinized, rehydrated, washed with H_2_O and stained with H&E to assess myocardial hypertrophy. Collagen distribution was assessed by staining with Masson trichrome and measuring the cardiomyocyte diameter using an Olympus fluorescence microscope (Olympus, Japan) under 200× magnification. A minimum of three random images was assayed per group and per assigned time.

### Real-time quantitative reverse transcriptase-**PCR (q**RT-PCR)

Total RNA from heart tissues (30 mg) or cultured NRCMs was separated using a Trizol reagent (Invitrogen, USA) in a homogenizer according to the manufacturer’s protocols. All procedures were performed according to previously reported protocols^31, 32^. The primers for ANP, β-MHC and GAPDH were described previously^1^. Real-time PCR was performed with quantitative PCR with SYBR Premix Ex Taq^TM^ (Takara, Japan). The samples were relatively quantified by normalizing to GAPDH, which served as an internal control.

### Western blotting analysis

GRP78, PERK, phosphorylated PERK, sXBP1, IRE1α, phosphorylated IRE1α, eIF2α, phosphorylated eIF2α, ATF6, CHOP, Bax, Bcl-2, caspase 3, 4-HNE, NOX 2 and NOX 4 were detected by western blotting analysis. In brief, heart tissues or cardiomyocyte lysates were prepared by homogenization with RIPA buffer. Total protein concentrations were measured using a BCA protein assay kit. Equal amounts of protein were separated by sodium dodecyl sulfate-polyacrylamide gel electrophoresis and transferred to polyvinylidene difluoride membranes (BIO-RAD, USA). The membranes were blocked with 5% nonfat milk powder prepared in Tris-buffered saline containing 0.1% Tween-20. Next, the membranes were incubated with primary antibodies overnight at 4 °C, followed by incubation with the appropriate horseradish peroxidase-linked secondary antibody. Finally, the immune complexes were detected using enhanced chemiluminescence and visualized with a Molecular Imager^R^ ChemiDoc^TM^ XRS+ System (Bio-Rad Laboratories, Hercules, CA, USA).

### Assessment of myocardial apoptosis

Cell apoptosis was assessed using fluorescein isothiocyanate (FITC)-labeled Annexin V and propidium iodide (PI) by flow cytometry as described previously^33, 34^. In brief, after high glucose or PA treatment with or without NAC, primary cardiomyocytes were digested with 0.125% trypsin and washed with PBS, followed by resuspension in 500 μL 1× binding buffer. Next, 5 μL Annexin V-FITC and 5 μL PI were added to each group in order. All samples were incubated on ice at 4 °C in the dark for 20 min. Finally, the mixtures were resuspended with a pipette and acquired immediately on the flow cytometer (BD Biosciences, CA).

### Detection of ROS generation in cardiomyocytes

To measure the total ROS induced by high-glucose or high-fat diets, NRCMs were treated with 30 mM glucose or 300 μM PA for 24 h and then incubated for 15 min with the fluorescent probe DCFH-DA (Sigma-Aldrich), which is a non-polar compound that can be taken up by cells and converted to a membrane-impermeable, non-fluorescent derivative by cellular esterases; finally, it can be oxidized to the fluorescent compound DCF by reactions with ROS. Mitochondrial ROS were detected using a MitoSOX Red superoxide indicator (Molecular Probes^TM^, USA) according to previous studies^23, 35^. Briefly, NRCMs were labeled with MitoSOX Red reagent, which fluoresces when oxidized by superoxide. After the stimulation with high glucose or PA in the presence or absence of apocynin (100 μM), an NAPDH oxidase inhibitor, or 10 μM of the mitochondria-targeting antioxidant Mito-TEMPO, cells were washed with 5 μM MitoSox Red solution in Hank’s Balanced Salt Solution for 10 min at 37 °C. The fluorescence was analyzed using an Operetta® High Content Imaging System (PerkinElmer, Massachusetts, USA). The fluorescence intensity of mitochondrial ROS was measured using ImageJ software.

### Statistical analysis

All data are shown as the mean±SEM and are analyzed by using Student’s *t*-test and multiple-comparison analysis of variance (ANOVA) in comparisons between two groups or multiple groups, respectively. *P* < 0.05 was considered to be statistically significant.

## Electronic supplementary material

Supplementary information
