# Supplementary material for: Palmitic acid, but not high-glucose, induced myocardial apoptosis is alleviated by N‑acetylcysteine due to attenuated mitochondrial-derived ROS accumulation-induced endoplasmic reticulum stress
Source: Cell Death Dis. 2018 May 11;9(5):568. doi: 10.1038/s41419-018-0593-y (PMC5948205; doi:10.1038/s41419-018-0593-y)

**Supplementary Methods**

**Determination of mitochondrial membrane potential (MMP)**

MMP levels were detected by mitochondrial membrane potential assay kit (#C2006, Beyotime Biotechnology, China) with 5, 50, 6, 60-tetrachloro-1, 10, 3, 30-tetraethylbenzimidazolylcarbocyanine iodide (JC-1) according to user manual. JC-1 is a fluorescent probe that can emit red fluorescence when concentrate in the mitochondrial matrix to form J-aggregates when MMP were high. Otherwise, JC-1 produces green fluorescence when MMP were low. A decrease in red/green ratio is indicative of a reduced MMP. According to this, neonatal rat cardiomyocytes (NRCMs) were grown in 96-well plate and treated with or without PA (300 μM) and CsA (0.5 μM). After 24 h exposure, the cells were washed with PBS and incubated with 2 μM of JC-1 dye in DMEM at 37°C for 20 min. The images were then taken by High Content Screening System (PerkinElmer, Massachusetts, USA) and the mitochondrial depolarization patterns of cells for quantification were examined using Operetta® High Content Imaging System (PerkinElmer, Massachusetts, USA).

**TUNEL assay**

The TUNEL method was performed to label 3′-end of fragmented DNA of the apoptotic NRCMs by One Step TUNEL Apoptosis Assay Kit (#C1086, Beyotime Biotechnology, China). The cells were grown in 96-well plate and treated with or without PA (300 μM), CsA (0.5 μM) and 4-PBA (1 mM). After 24 h exposure, NRCMs were fixed with 4% paraform phosphate buffer saline, rinsed with PBS, then permeabilized by 0.1% Triton X-100 for FITC end-labeling the fragmented DNA of the apoptotic PASMCs using TUNEL cell apoptosis detection kit. The FITC-labeled TUNEL-positive cells were imaged by High Content Screening System (PerkinElmer, Massachusetts, USA).

**Supplemental Figure Legends**

**Supplemental Figure 1: CsA attenuates PA-induced cardiomyocytes apoptosis and ER stress.** NRCMs were isolated and cultured by treatment with or without PA (300 μM) in the presence or absence of CsA (0.5 μM), an inhibitor of mitochondrial membrane potential. (A) Photograph showing JC-1 red, JC-1 green and merge image; Scale bar, 50 μm. And numerical data were expressed in terms of the ratio of JC-1 aggregates to JC-1 monomers. (B) The TUNEL assay was carried out by One Step TUNEL Apoptosis Assay Kit. The images of TUNEL positive cells were captured by a fluorescence microscope; Scale bar, 100 μm. (C) Representative western blot analysis of Bcl-2, Bax and cleaved caspase 3 in NRCMs after treatment with PA (300 μM) with or without CsA (0.5 μM). (D) ER stress-associated signaling pathways, such as GRP 78, p-IRE1α/IRE1α, p-eIF2α/eIF2α, p-PERK/PERK and CHOP were also examined by western blotting in NRCMs. GAPDH was used as a loading control. ImageJ software was used to measure the band intensity, and each band intensity was normalized to GAPDH. All data are presented as the mean ± SEM of three independent experiments. **P* <0.05, ***P* <0.01 vs control group; ^#^*P* <0.05, ^##^*P* <0.01 vs PA-treated NRCMs.

**Supplemental Figure 2: 4-PBA suppresses PA-induced cardiomyocytes apoptosis.** NRCMs were isolated and cultured by treatment with or without PA (300 μM) in the presence or absence of 4-PBA (1 mM), an inhibitor of ER stress. (A) The TUNEL assay was carried out by One Step TUNEL Apoptosis Assay Kit. The images of TUNEL positive cells were captured by a fluorescence microscope; Scale bar, 100 μm. (B) Representative western blot analysis of Bcl-2, Bax and cleaved caspase 3 in NRCMs after treatment with PA (300 μM) with or without 4-PBA (1 mM). GAPDH was used as a loading control. ImageJ software was used to measure the band intensity, and each band intensity was normalized to GAPDH. All data are presented as the mean ± SEM of three independent experiments. ***P* <0.01 vs control group; ^##^*P* <0.01 vs PA-treated NRCMs.

**Supplemental Figure 1**


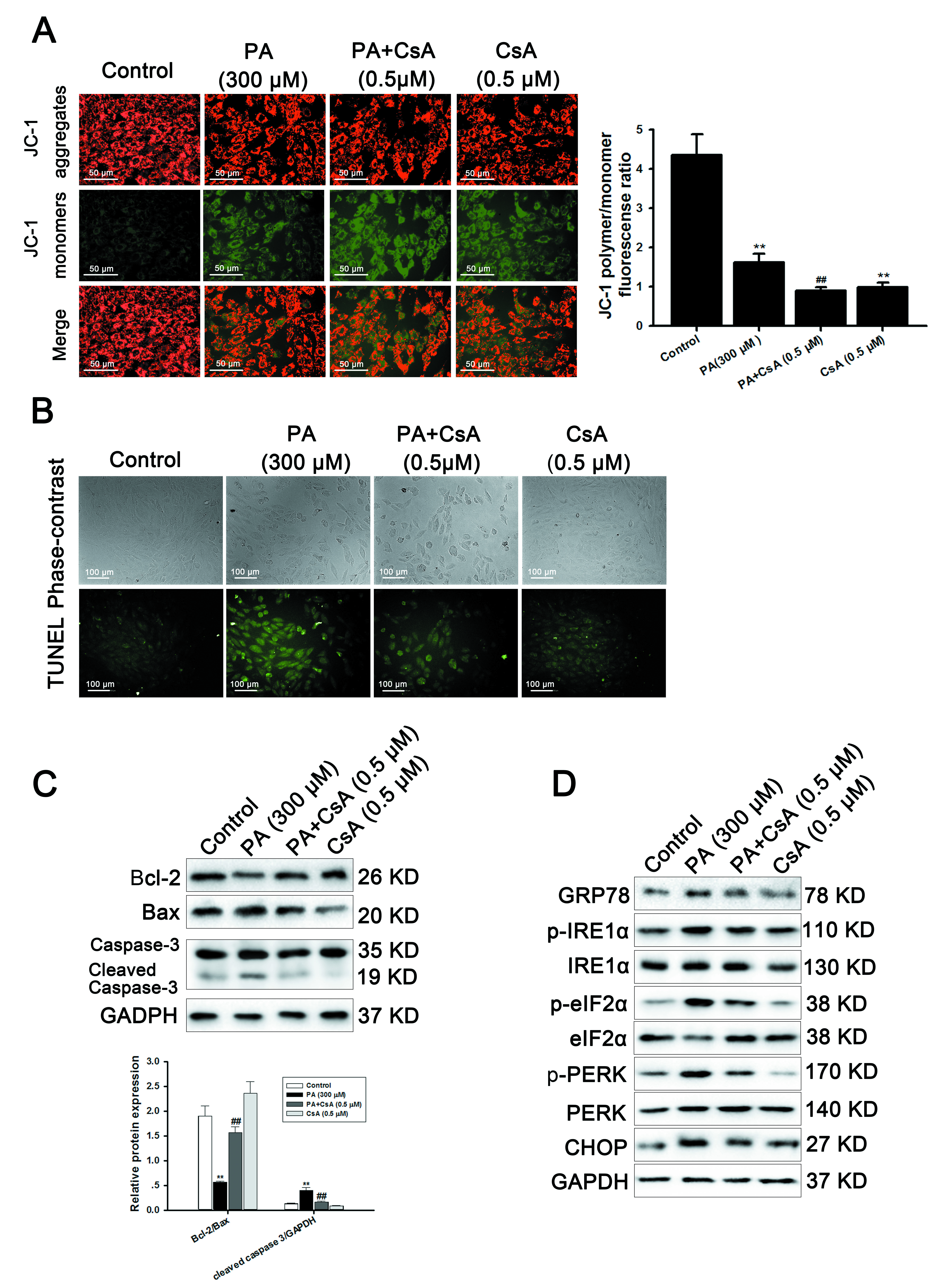


**Supplemental Figure 2 (Figure S2)**


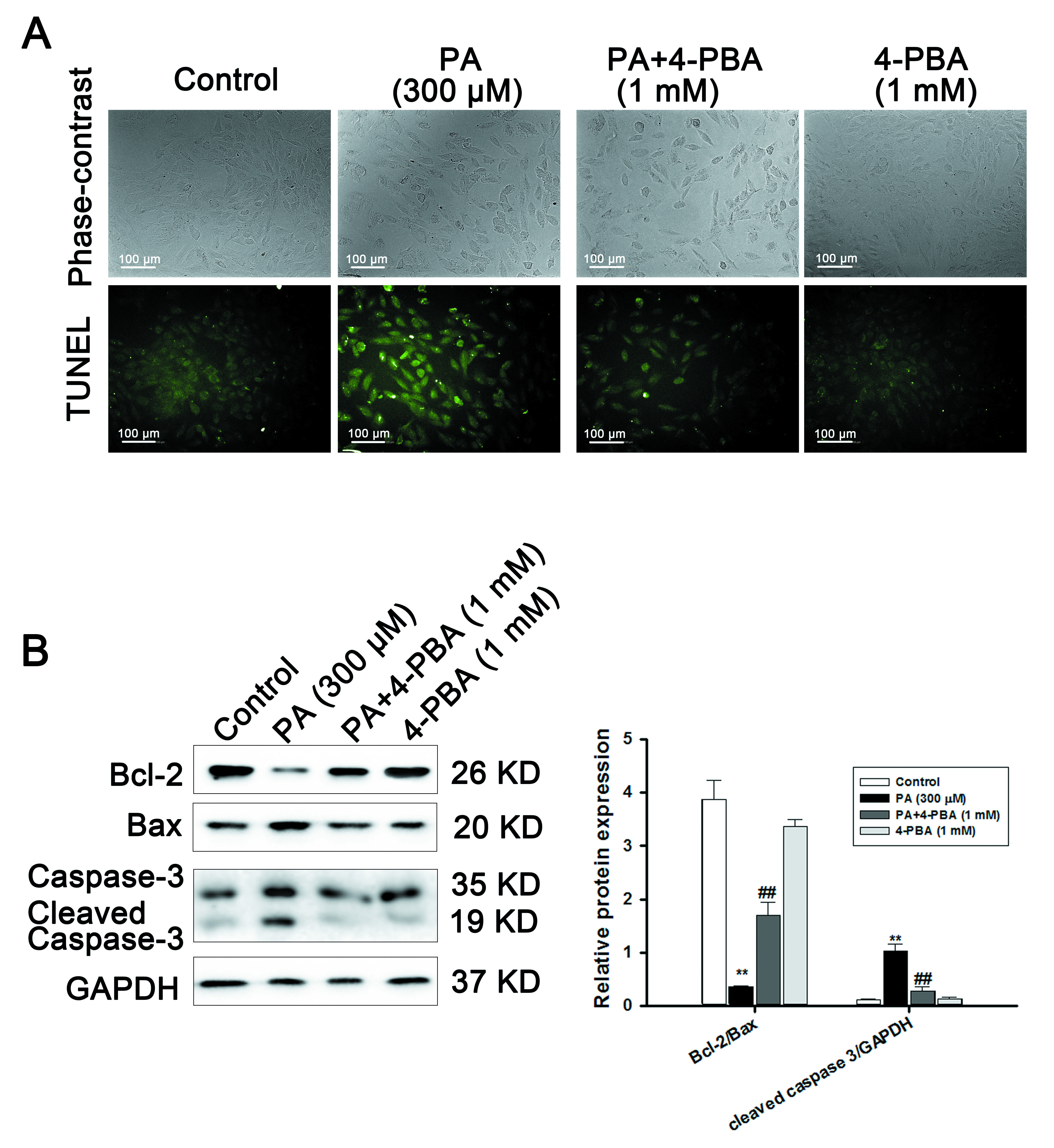

Supplement: Supplementary file 1 — Supplementary information [file 41419_2018_593_MOESM1_ESM.docx]
